# Impact of different gutta-percha removal techniques on dentinal integrity: An *in vitro* analysis

**DOI:** 10.4317/jced.62524

**Published:** 2025-02-01

**Authors:** Orlando Aguirre Guedes, Henrique Carneiro Ferreira, Danielly Moreira Abreu, Kássia Sousa de Lima, Esther Menezes Souza, Yasmin Gomes de Freitas, Daniel de Almeida Decurcio, Carlos Estrela

**Affiliations:** 1 Department of Endodontics, School of Dentistry, Evangelical University of Goiás, Anápolis, GO, Brazil; 2 Department of Stomatology Sciences, School of Dentistry, Federal University of Goiás, Goiânia, GO, Brazil

## Abstract

**Background:**

This study aimed to evaluate the impact of different gutta-percha removal (GPR) techniques on the occurrence of dentinal defects in bovine root canals.

**Material and Methods:**

One hundred and eight bovine incisors were selected and stored in distilled water. The crowns were removed, leaving roots approximately 17 mm in length. Twelve roots were left unprepared and served as control (G1), and the remaining 96 roots were instrumented with BioRace files up to size 40/.04 (BR5) and filled with gutta-percha and Sealapex sealer using the lateral condensation technique. Twelve other roots were left filled and received no retreatment procedure (G2). The remaining 84 roots underwent filling material removal with R-Endo (G3), D-Race (G4), WaveOne Gold (G5), ProTaper Retreatment (G6), Mtwo R (G7), Reciproc (G8) instruments or with R1-Clearsonic ultrasonic insert (G9). After GPR, final apical enlargement was achieved using a BR7 (#60/.02) instrument. Roots were sectioned 3, 6 and 9 mm from the apex and observed under a stereomicroscope at 25ᵡ magnification. The presence/absence of root fractures, microcracks, and craze lines were recorded. Chi-square tests compared the incidence of dentinal defects between the groups, with a significance level set at *P*<.05.

**Results:**

Defects occurred in 46.6% of the samples. No defects were observed in the unprepared canals (control, G1). Defects were detected in all other experimental groups (G2 to G9). Among retreatment techniques the R-Endo group (G3) presented significantly higher microcracks and craze lines (*P*<.05). Defects were more frequent in the coronal and middle thirds.

**Conclusions:**

All GPR methods were linked to dentinal defects. The R-Endo system significantly increased microcracks and craze lines.

** Key words:**Cracks, Dentinal defects, Gutta-percha removal, Retreatment, Vertical root fracture.

## Introduction

Signs of root canal treatment failure, such as the presence of apical periodontitis or persistent post-treatment symptoms, indicate the need for further intervention (1). Nonsurgical root canal retreatment is often recommended when the initial treatment fails to achieve success (2-5). Retreatment success depends on efficient removal of previous filling materials, which allows for re-shaping, disinfection, and refilling of the root canal system (2,6,7).

Various niquel-titanium (NiTi) systems with different tapers, cutting blades and tip conFigurations, have been developed to enhance the efficiency of gutta-percha removal (4,5,7). Notable systems include Mtwo-R® (VDW, Munich, Germany), D-RaCe® (FKG Dentaire, La Chaux-de-Fonds, Switzerland), R-Endo® (Micro-Mega, Besançon, France) and ProTaper Universal Retreatment® (Dentsply Maillefer, Ballaigues, Switzerland). These rotary instruments are specifically designed for retreatment procedures (2,4-9).

In recent years, additional tools have been introduced into the nonsurgical root canal retreatment arsenal (9-12). Instruments such as Reciproc® (VDW, Munich, Germany) and WaveOne® (Dentsply Maillefer, Ballaigues, Switzerland), originally developed for root canal preparation (13,14), are now also used for the removal of filling material (12,15). Another innovative device is the R1-Clearsonic® ultrasonic insert (Helse Ultrasonic, Santa Rosa de Viterbo, SP, Brazil), specifically engineered for retreatment purposes (11). Both reciprocating and ultrasonic techniques have shown satisfactory performances in gutta-percha and sealer removal (10,12,15).

Stresses generated within the root structure can be transmitted to its surface, potentially disrupting the bonds that hold the dentin together and leading to the formation of microcracks (16). The impact of endodontic and restorative procedures on the dentin integrity is a significant concern (1,6,14) . The use of larger or stiffer instruments increases contact with the canal walls, resulting in greater friction and stress concentration, which may contribute to the development of dentinal defects (4,5).

Cracks lines and microcracks formed during endodontic and restorative procedures can propagate over time due to subsequent interventions or repeated occlusal forces, eventually leading to vertical root fractures (VRF) (6,17,18). VRF are considered the most common cause for the tooth loss in root canal-treated teeth (3,13,14,17,19) and should therefore be actively prevented (1,3,9,13,18,20). A potential prevention strategy involves identifying and comparing procedures that are most likely to induce dentin defects (2,18).

Although *in vitro* studies have demonstrated that various intraradicular procedures - such as root canal instrumentation, obturation, post space preparation, and post removal - can contribute to the development of dentinal defects on the root canal walls (17,20-23), limited attention has been given to the occurrence of root fractures and other defects specifically after gutta-percha removal (GPR) (1,4-7,9,18). Root canal retreatment involves additional mechanical manipulation and further preparation of the canal (4,6-8), which often results in the removal of more dentin tissue from the root canal walls (6). Consequently, the likelihood of dentinal defects may increase following these procedures (4,5).

A comprehensive literature review revealed that the impact of reciprocating instruments on dentin following retreatment procedures has been examined in only one study (9). Notably, no studies have evaluated the effects of WaveOne® instruments. Additionally, it remains unclear whether the use of R1-Clearsonic® ultrasonic insert may lead to greater damage to the root canal walls. Therefore, this study aimed to assess the effects of different GPR techniques on the occurrence of dentinal defects. The null hypothesis tested was that the occurrence of root fractures and other dentin defects does not vary based on (i) the retreatment protocol used and (ii) the root canal level.

## Material and Methods

The present study protocol was approved by the Institutional Animal Care and Use Committee (#001/2021).

-Sample size calculation

The sample size was calculated based on effect size estimations of dentinal defects induced by root canal instrumentation and filling, as reported by Shemesh *et al*. (7), and by gutta-percha removal, as described by Yilmaz *et al*. (18). Using an alpha error probability of 0.05 and a power of 80%, the software G*Power 3.1.2 (Heinrich Heine University Düsseldorf, Düsseldorf, Germany) determined that a sample size of 12 teeth per group was required.

-Sample selection and preparation

Freshly extracted bovine incisors with fully formed roots, similar in size and shape, and exhibiting straight, single canals of comparable widths were selected. Canal widths were measured 9 mm from the apex using preoperative radiographs taken from both bucco-lingual and mesio-distal directions (9,17,22). Teeth with curved roots, calcified or flared root canals, or significant anatomical irregularities were excluded from the study. A total of 108 teeth met the inclusion criteria and were stored in distilled water at 4oC until use.

The crowns of the teeth were sectioned using a double-faced diamond disc (KG Sorensen, São Paulo, SP, Brazil) positioned perpendicularly to the longitudinal axis of the teeth, standardizing the root length at 17 mm. The external root surfaces were examined under 20ᵡ magnification using a stereomicroscope (Expert DN; Mϋller Optronic, Erfurt, Germany) to detect preexisting defects. Roots exhibiting cracks, fractures, or craze lines were excluded and replaced with similar specimens.

To simulate the periodontal ligament space, the roots were coated with silicone impression material (Aquasil, Dentsply Maillefer, Ballaigues, Switzerland) (17,24) and embedded in self-curing acrylic resin within a cylindrical tube (9). Twelve roots were left unprepared to serve as controls (Group 1), while the remaining 96 roots underwent root canal instrumentation.

-Root canal instrumentation

Apical patency of the root canals was confirmed using a #10 K-File (Dentsply Maillefer, Ballaigues, Switzerland). To ensure standardization, roots with patency larger than an ISO #15 K-File (Dentsply Maillefer, Ballaigues, Switzerland) were replaced (21,23). All roots were instrumented to a work length (WL) of 16 mm (1 mm short of the apex) using a crown-down technique with BioRace rotary instruments (FKG Dentaire, La Chaux-de-Fonds, Switzerland) using a torque and speed-controlled motor (X-Smart Plus; Dentsply Maillefer, Ballaigues, Switzerland). The full BioRace Basic Set (FKG Dentaire, La Chaux-de-Fonds, Switzerland) was used at 600 rpm and torque of 1 Ncm for canal preparation, following the sequence: BRO (#25/.08), BR1 (#15/.05), BR2 (#25/.04), BR3 (#25/.06), BR4 (#35/.04) and BR5 (#40/.04) (22). Each new instrument set was used to prepare three root canals.

During instrumentation, roots canals were irrigated with 3 mL of 1.0% sodium hypochlorite solution (NaOCl; Fitofarma, Goiânia, GO, Brazil) delivered with a syringe and a 31-gauge needle (NaviTip, Ultradent, South Jordan, UT, USA) after each file change. Following root canal preparation, the canals were flushed with 3 mL of 17% EDTA (Biodinâmica, Ibiporã, PR, Brazil) for 3 min, followed by 3 mL of 1.0% NaOCl (Fitofarma, Goiânia, GO, Brazil). Finally, the external root surfaces were re-examined for defects under 20ᵡ magnification using a stereomicroscope (Expert DN; Mϋller Optronic, Erfurt, Germany), and no visible defects were detected.

-Root canal obturation

Before obturation, the roots canals were dried with sterilized paper points (Dentsply Maillefer, Ballaigues, Switzerland). All canals were filled using the lateral condensation technique. Gutta-percha cones (Dentsply Maillerfer, Ballaigues, Switzerland) were coated with Sealapex Sealer (SybronEndo, São Paulo, SP, Brazil) and inserted into the root canal to the WL. Accessory gutta-percha cones were added using a size B spreader (Dentsply Maillerfer, Ballaigues, Switzerland).

Excess gutta-percha and sealer were removed with flame-heated vertical condensers, and access cavities were sealed with a temporary restorative material (Vidrion R, SS White, Rio de Janeiro, RJ, Brazil). Radiographs were taken in bucco-lingual and mesio-distal angles to confirm the quality of the obturation. Specimens with inadequate obturation were replaced.

The external roots surfaces were re-examined for defects under 20ᵡ magnification using a stereomicroscope (Expert DN; Mϋller Optronic, Erfurt, Germany), and no visible defects were detected. The specimens were then immersed in distilled water at 37oC for 7 days to allow the sealer to set (17). Of the 96 obturated roots, 12 were left filled without no retreatment and assigned to Group 2.

-Gutta-percha removal methods

The remaining 84 roots were randomly divided into 7 groups (n = 12). Root fillings materials were removed using the following methods:

-R-Endo group (Group 3)

In this group, root canal filling material was removed using R-Endo instruments (Micro-Mega, Besançon, France) following a specific sequence. First, the Rm stainless steel hand file (#25/.04) was applied with 1/4 turn pressure directed toward the apex to create a pathway for centering and aligning the subsequent rotary instruments. The rotary instruments were operated at a speed of 350 rpm. The Re instrument (#25/.12) was used to remove the first 2-3 mm of filling material, followed by R1 (#25/.08) and R2 (#25/.06) instruments, which advanced to one-third and two-thirds to the WL, respectively. Finally, the R3 (#25/.04) and Rs (#30/.04) instruments were employed at the WL using a circumferential filling motion from the apical to the coronal third (2,4). A new set of instruments was used for every three root canals.

-D-RaCe group (Group 4)

In this group, root canal fillings were removed using the D-RaCe retreatment instruments (FKG Dentaire, La Chaux-de-Fonds, Switzerland). The procedure began with the DR1 instrument (#30/.10) at a speed of 1000 rpm and a torque of 1.5 Ncm to prepare the cervical third and the initial portion of the middle third. This was followed by the DR2 instrument (#25/.04) at a speed of 600 rpm and a torque of 1 Ncm, which was used to reach the WL. The DR2 instrument was applied with light apical pressure until the WL was achieved (4). A new set of instruments was used for every three root canals.

-Wave One group (Group 5)

In this group, root filling material was removed using the Wave One primary instrument (#25/.07) (Dentsply Maillefer, Ballaigues, Switzerland). The instrument was operated with an endodontic motor (X-Smart Plus, Dentsply Maillefer, Ballaigues, Switzerland), utilizing an in-and-out pecking motion with an amplitude of approximately 3 mm. The “WAVEONE ALL” program was used until the WL was achieved. During the procedure, gentle apical pressure was applied, and a brushing was used to facilitate filling material removal (15). A new set of instruments was used for every three root canals.

-ProTaper Universal Retreatment group (Group 6)

In this group, root canal filling material was removed using the ProTaper Universal Retreatment instruments (Dentsply Maillefer, Ballaigues, Switzerland). The D1 instrument (#30/.09) was employed to remove filling material from the coronal third, followed by the D2 instrument (#25/.08) for the middle third. The D3 instrument (#20/.07) was used to reach the full WL. Files progression was performed with slight apical pressure and amplitude of no more than 3 mm. The instruments were operated at a constant speed of 500 rpm for D1 and 400 rpm for D2 and D3, with a torque of 3 Ncm (4). A new set of instruments was used for every three root canals.

-Mtwo-R group (Group 7)

In this group, root filling material was removed using the Mtwo R2 instrument (#25/.05) (VDW, Munich, Germany) operated at a speed of 280 rpm and a torque of 1.2 Ncm. A brushing motion was applied against the root canals walls in a crown-down direction until the WL was achieved (4,6). A new set of instruments was used for every three root canals.

-Reciproc group (Group 8)

In this group, root filling material was removed using the Reciproc R25 instrument (#25/.08) (VDW, Munich, Germany). The instrument was operated with an endodontic motor (X-Smart Plus, Dentsply Maillefer, Ballaigues, Switzerland), utilizing an in-and-out pecking motion with an amplitude of approximately 3 mm. The “RECIPROC ALL” program was followed until the WL was achieved. Gentle apical pressure was applied during the procedure, along with a brushing motion to facilitate the removal of filling material (9). A new set of instruments was used for every three root canals.

-R1-Clearsonic group (Group 9)

In this group, root canal filling was removed using the R1-Clearsonic® ultrasonic insert (Helse Ultrasonic, Santa Rosa de Viterbo, SP, Brazil), activated by the EMS PM 200 ultrasonic unit (EMS - Electro Medical Systems S.A., Nyon, Switzerland). The ultrasonic unit was set to 30% power. The ultrasonic insert was advanced to the WL and activated, performing continuous in-and-out movements against the root canal walls (10).

All rotary NiTi instruments were operated with a torque and speed-controlled motor (X-Smart, Dentsply Maillefer, Ballaigues, Switzerland), following the manufacturer’s recommended settings for each system. To complete the GPR, additional preparation was performed with BioRace Extended Set instruments (FKG Dentaire, La Chaux-de-Fonds, Switzerland) up to size BR7 (#60/.02), maintaining the same speed and torque values previously described. A new set of instruments was used for every three root canals.

During the retreatment, root canals were irrigated with 3 mL of 1.0% NaOCl (Fitofarma, Goiânia, GO, Brazil) after each file change. Final irrigation consisted of 3 mL of 17% EDTA (Biodinâmica, Ibiporã, PR, Brazil) for 3 min, followed by 3 mL of 1.0% NaOCl (Fitofarma, Goiânia, GO, Brazil). Retreatment was considered complete when no debris of root filling materials were detected on the instrument surfaces or in the irrigating solution (17). The smoothness of canal walls was assessed using tactile sensitivity with the last instrument. All root canal instrumentation, filling and retreatment procedures were performed by a single operator, an endodontist with more than 10 years of experience. Specimens were stored in distilled water throughout the study to prevent dehydration (4,5).

-Root canal sectioning, staining, and stereomicroscopic examination

All roots were removed from the resin blocks, had the silicone impression material removed, and were horizontally sectioned at 3, 6 and 9 mm from the root apex. Sectioning was performed using a double-faced diamond disc (4” diameter ᵡ 0.012” thickness ᵡ 1/2”; Arbor, Extec, Enfield, CT, USA) mounted on a precision saw (Isomet 1000, Buehler, Lake Bluff, IL USA) at low speed with water cooling.

The slices were air-dried with absorbent paper, stained with 1% methylene blue to aid defect detection (21,22), rinsed with distilled water, dried again, and examined under a 25ᵡ magnification using a stereomicroscope (Expert DN; Mϋller Optronic, Erfurt, Germany). Digital images of all slices were captured with a camera attached to the stereomicroscope.

The images were analyzed, and the presence of defects was registered as “no defect”, “fracture”, and “all other defects” (3).

**Figure 1:**
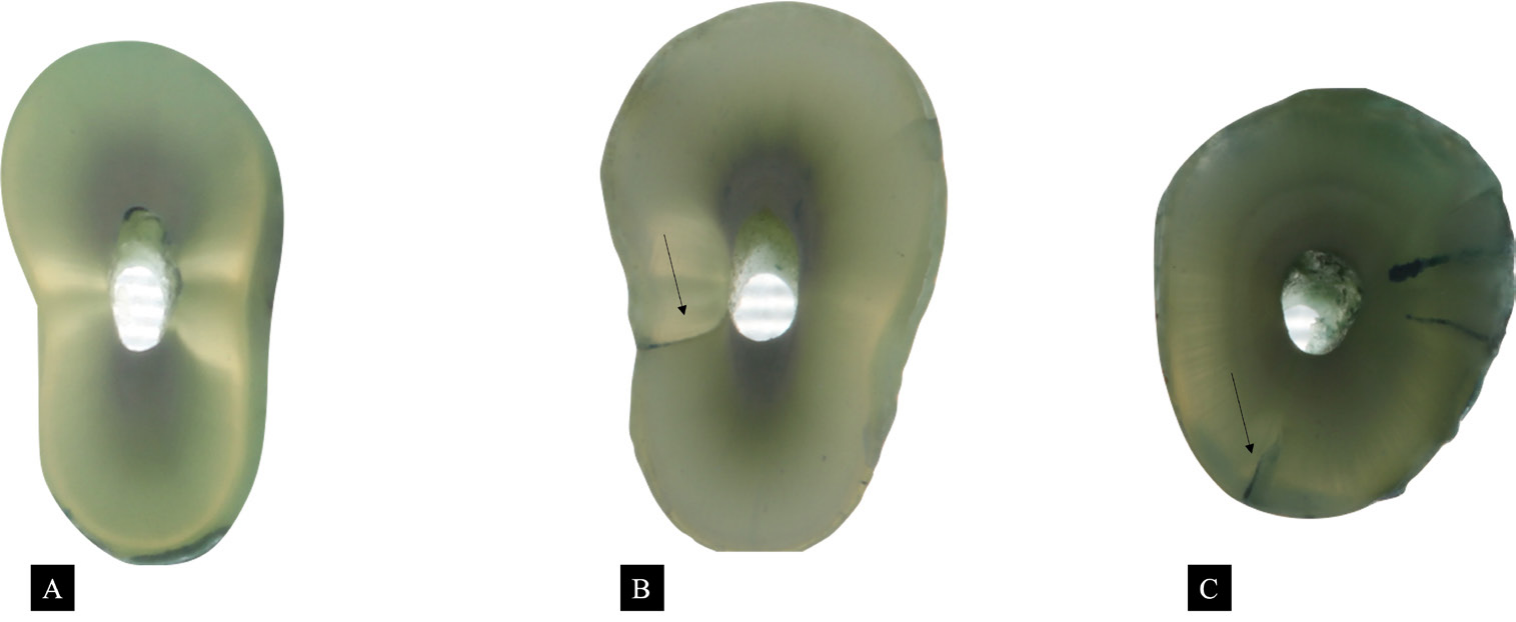
Representative images of dentin slices: (A) Dentin slice without defects; (B) Dentin slice showing a root fracture (indicated by a black arrow); (C) Dentin slice showing a partial crack (indicated by black arrows).

“No defect”: Root dentin without any craze lines or microcracks, where both the external root surface and the internal canal wall showed no defects (Fig. 1A).

“Fracture”: A line extending from the root canal space to the outer root surface (Fig. 1B).

“All other defects”: Partial cracks (extending from the root canal wall into the dentin without reaching the outer surface) and craze lines (extending from the outer surface into the dentin but not reaching the canal lumen or from the outer surface or canal wall into dentin) (Fig. 1C).

A total of 324 images (36 images per group) were analyzed. An examiner blinded to the retreatment protocol analyzed all the images after calibration. Each image was reviewed twice, with a 14-day interval between the readings.

-Statistical analysis

The results were presented as the number and percentage of defects observed in each group. Statistical analyses were performed using IBM SPSS for Windows, version 21.0 (IBM Corp., Armonk, NY, USA). The Chi-square test was applied to assess differences between groups and to evaluate the influence of root canal levels on the development of dentinal defects. The significance level for all analyses was set at *P*<.05.

Multiple comparisons were conducted using the z-test with Bonferroni correction. Intraexaminer agreement was evaluated using Cohen’s kappa test to ensure consistency and reliability.

## Results

The Cohen’s kappa coefficient was 0.91, indicating excellent intraexaminer reliability. The distribution of dentinal defects across the groups is summarized in Tables 1 and 2. Of the 324 dentin discs evaluated, 151 (46.6%) exhibited some form of root defect. Among these, 108 (71.5%) were classified as all other defects, while 43 (28.5%) were specifically identified as root fractures.

No defects were observed in the unprepared canals (control group, G1), whereas all experimental groups exhibited the presence of defects. Comparing the filled but unretreated group (G2) with the retreated groups, no statistically significance difference was found in the frequency of root fractures (*P*>.05) ( Table 1). Similarly, no significant differences were detected among the GPR protocols regarding root fractures (*P*>.05) ( Table 1).

All GPR protocols resulted in a higher incidence of partial cracks and craze lines compared to the unretreated group. However, a statistically significant difference was observed only for the R-Endo group (G5) (*P*<.05). No statistically significant differences were observed between the GPR groups in terms of “all other defects” (*P*>.05) ( Table 2).

When analyzing the distribution of defects at different evaluation levels (3, 6 and 9 mm from the apex), significant differences were noted in the occurrence of root fractures (*P* = 0.034) (T Table 1). A greater number of root fractures were detected in sections taken at 9 mm and at 6 mm from the apex ( Table 1).

## Discussion

This *in vitro* study aimed to evaluate whether the method of gutta-percha removal (GPR) used during retreatment procedures influences the incidence of dentinal defects in the root canals. Root filling material was removed using the commonly utilized systems such as R-Endo®, D-Race®, ProTaper Universal®, and Mtwo-R® systems, along with more recently introduced systems, including Reciproc® and WaveOne®, as well as the ultrasonic R-1 Clearsonic®. To date, no prior study has evaluated the effects of WaveOne® and R-1 Clearsonic® on the formation of dentinal defects. The findings of this study revealed that the root canal level was the only factor significantly influencing the occurrence of root dentin defects. Consequently, the null hypotheses were partially rejected.

Various methodologies have been developed to assess the impact of endodontic and restorative procedures on root dentin integrity (8,16,25). Among these, the tooth-sectioning method used in the present study widely employed (3,9,13,16,21-23) due to its ability to facilitate direct inspection of dentin discs obtained from different levels (3,13). Despite its advantages, this method is destructive, limiting subsequent analyses (1,16,18,25-27). Additionally, its inability to precisely determine when defects occurs (9) has driven the exploration of alternative methodologies (16,25-27). Yilmaz *et al*. (18) employed micro-CT imaging to investigate the incidence and progression of dentin defects following gutta-percha removal using hand and rotary instruments. This non-destructive approach enables both quantitative and qualitative volumetric assessments of teeth. By comparing pre- and postoperative images, the preoperative state served as an authentic control (2,8). However, the limitations of micro-CT, including limited availability, high costs, and time-consuming analysis, can restrict its broader application (23).

In the current study, the roots of the selected teeth were examined under a stereomicroscope prior to the experiment to detect any external root defects. It is acknowledged that some defects might have been internal and not visible on the outer root surface. However, no defects were observed in the control group (unprepared roots), indicating that the sectioning method itself did not induce defects (1,3,14). This finding suggests that the observed defects were most likely caused by procedures such as root canal instrumentation, filling, and gutta-percha removal, rather than the tooth-sectioning technique (5,14).

Previous studies have utilized teeth with varying root canal morphologies, including mandibular incisors (1,18,19), and mandibular premolars (2,4-7,9). This variability in anatomy complicates the comparison of results across studies. It has been proposed that root and canal morphology may influence the occurrence of root dentin defects (13,14,21). To address these challenges, the present study employed bovine teeth, which are easier to obtain, allow for better standardization of age and canal space, and reduce the risk of transmitting infectious diseases (22). Despite some microstructural and macrostructural differences between human and bovine teeth, several studies have deemed bovine teeth an appropriate model for this type of research (22,23). Older bovine teeth were preferred in this study due to their potential for greater morphologic resemblance to human teeth (22). Additionally, the roots were evenly distributed among the groups based on their root canal diameter at the 9-mm level (9,17,22). Standardization within the groups was ensured by selecting roots with apical patency compatible with a size 15 K-file (21,23) and maintaining an approximate root length of 17 mm.

It has been demonstrated that specimen storage conditions can significantly affect the biochemical properties of root dentin (4,5). In the present study, all specimens were stored in distilled water at 37oC throughout the entire experiment period, in accordance with previous recommendations (21,26).

To simulate the periodontal ligament and enhance the clinical relevance of the results, the roots in the present study were surrounded by a silicone-based material and embedded in acrylic resin blocks (3,21,24,28). It is important to note that, to date, there is no standardized experimental design for effectively simulating the periodontal ligament (3,14,21). Furthermore, the clinical condition is more complex due to the presence of additional biological structures that are challenging to replicate in a laboratory setting (13,24). A recent study assessed the formation of dentin cracks following root canal preparation using an in situ cadaver model (27). This methodological approach provided greater reproducibility for studying dentin defects by preserving both bone and the periodontal ligament.

The effect of rotary and reciprocating system on the developmental of dentinal defects during root canal instrumentation has been widely reported in the literature (13,14,17,20,21). In some studies, no significance differences in defect formation were observed, regardless of the type of instrument used (21). In this study, initial root canal instrumentation was performed using the BioRace Basic Set, which consists of six instruments: BR0 (#25/.08), BR1 (#15/.05), BR2 (#25/.04), BR3 (#25/.06), BR4 (#35/.04) and BR5 (#40/.04). The BioRace system was selected due to its alternating cutting angle, inactive tip, triangular cross-sectional design without radial bands, and electrolytic surface treatment (29). These features have been associated with a reduced incidence of dentinal defects (29). It is noteworthy that the BR0 and BR3 files have a high taper (.08 and .06, respectively), and tapered instruments may increase contact area with canal walls, creating localized stress concentration in the dentin. This could contribute to the development of dentinal defects (17,18,21). Additionally, the BioRace system operates in continuous rotation (29), requiring a greater number of rotations to complete the root preparation (5). Increased rotations inside the canal may lead to greater friction between the instrument and the canal walls, potentially resulting in a higher incidence of dentinal defects (2,5,14,18).

Despite the extensive investigations into the influence of obturation procedures on the formation of dentinal defects (3,26,28,30), the findings remain inconsistent, particularly regarding the impact of the lateral condensation technique (3,19,26,28). Shemesh *et al*. (3) compared the occurrence of dentinal defects following obturation using lateral condensation versus passive condensation techniques, reporting a higher incidence of defects in teeth obturated with lateral condensation method. Similarly, Capar *et al*. (19) examined the incidence of cracks in root dentin after filling with cold lateral condensation, single-cone obturation, and warm vertical compaction. Their results indicated that both warm vertical and cold lateral compaction techniques resulted in a greater number of cracks compared to single-cone obturation. In a related study, Adorno *et al*. (30) investigated the effects of root preparation and obturation methods (lateral condensation with or without vertical compaction) on crack initiation and propagation in apical third of the root. They found that while the obturation procedure significantly influenced crack propagation, there was no significant difference in crack initiation between the two techniques. Furthermore, Shemesh *et al*. (28) assessed the incidence of dentinal defects after canal preparation and obturation using lateral condensation versus continuous wave compaction of gutta-percha, finding no significant differences in defect occurrence between the two methods. In contrast, De-Deus *et al*. (26) concluded that the cold lateral condensation was not associated with the development of new root defects.

In the current study, the lateral condensation technique was chosen for obturation due to its widespread acceptance in clinical practice, the absence of a need for specialized or costly equipment, and its effectiveness in controlling the apical extension of the filling material (3,18,28). However, it is important to consider that the design of the spreader and the excessive pressure applied during lateral compaction can significantly contribute to the formation of dentinal defects (3,9,18-20). Notably, the findings of this study revealed a lower incidence of defects (5.3%) compared to previous research, which reported detect rates ranging from 16 to 30% following root canal preparation and filling (26,28). This discrepancy may be attributed to variations in study methodologies (16,25), highlighting the need for standardized protocols in future investigations.

The present study assessed the formation of dentinal defects associated with seven different GPR methods. The selection of NiTi systems and the ultrasonic insert for root filling material removal was based on prior studies demonstrating the efficiency of these techniques (10-12). All tested GRP methods were found to induce dentinal defects ( Table 1, Table 2). Notably, GPR using R-Endo system (Group 3) showed a significant impact on the development of microcracks and craze lines when compared to unretreated group (Group 2) ( Table 2). The highly-tapered design of R-Endo instruments (12% and 8%) may explain the increased susceptibility to “all other defects” formation (2).

Previous studies have reported that the characteristics of NiTi instrumentation files, such as tip design, cross-section shape, variable or constant taper and type of movement, may influence the formation of dentinal defects (1,8,24). However, the results of the present study did not show significant differences among GPR methods regarding the presence of root defects. This suggests that variations in GPR instrument design did not significantly impact the formation of dentinal defects (1,4).

Although dentinal defects were observed at all 3 levels of the root canal, a higher number of root fractures, microcracks and craze lines were found in the coronal sections (9 and 6 mm) compared to the apical sections (3 mm) ( Table 1, Table 2). This finding aligns with the results of previous studies (8,19,21,23). The increased incidence of coronal dentin defects may suggest the possibility of excessive tapering in the coronal third (9). However, the *in vitro* nature of the present study poses limitations for clinical extrapolation. Further clinical studies are needed to evaluate the advantages of different GPR protocols more comprehensively.

## Conclusions

Under the conditions of this *in vitro* study, it may be concluded that: 1. All GPR methods induced dentinal defects during retreatment procedures; 2. GPR with the R-Endo system was associated with significantly more microcracks and craze lines; and 3. Root fractures were more frequently observed in the 9-mm and 6-mm sections.

## Figures and Tables

**Table 1:** Number and percentage of root fractures in the different cross-section slices.

Groups	Root level			Total	P value*
	9 mm	6 mm	3 mm		
G1	0 (0%)^A,a^	0 (0%)^A,a^	0 (0%)^A,a^	0 (0%)^A^	> 0.05
G2	1 (2.3%)^A,a^	1 (2.3%)^A,a^	1 (2.3%)^A,a^	3 (2.8%)^AB^	1.000
G3	3 (7.0%)^A,a^	3 (7.0%)^A,a^	1 (2.3%)^A,a^	7 (6.5%)^AB^	0.492
G4	2 (4.7%)^A,a^	2 (4.7%)^A,a^	1 (2.3%)^A,a^	5 (4.6%)^AB^	0.793
G5	3 (7.0%)^A,a^	1 (2.3%)^A,a^	1 (2.3%)^A,a^	5 (4.6%)^AB^	0.395
G6	0 (0%)^A,a^	1 (2.3%)^A,a^	0 (0%)^A,a^	1 (0.9%)^AB^	0.373
G7	3 (7.0%)^A,a^	3 (7.0%)^A,a^	2 (4.7%)^A,a^	8 (7.4%)^AB^	0.852
G8	4 (9.3%)^A,a^	4 (9.3%)^A,a^	1 (2.3%)^A,a^	9 (8.3%)^B^	0.264
G9	3 (7.0%)^A,a^	2 (4.7%)^A,a^	0 (0%)^A,a^	5 (4.6%)^AB^	0.197
Total	19 (44.2%)^a^	17 (39.5%)^a,b^	7 (16.3%)^b^	43 (100%)	0.034
P Value*	0.308	0.424	0.769	0.025	

*Chi-square test. G1: Control; G2: Instrumentation and filling; G3: R-Endo; G4: D-Race; G5: WaveOne Gold; G6: ProTaper Retreatment; G7: Mtwo R; G8: Reciproc; G9: R1-Clearsonic. Capital letters compare groups in vertical columns and lower-case letters compare groups in horizontal rows.

**Table 2:** Number and percentage of all other defects in the different cross-section slices.

Groups	Root level			Total	P value*
	9 mm	6 mm	3 mm		
G1	0 (0%)^A,a^	0 (0%)^A,a^	0 (0%)^A,a^	0 (0%)^Aa^	> 0.05
G2	3 (2.8%)^A,a^	1 (0.9%)^AB,a^	1 (0.9%)^A,a^	5 (4.6%)^A,B^	0.395
G3	6 (5.6%)^A,a^	8 (7.4%)^B,a^	5 (4.6%)^A,a^	19 (17.6%)^C^	0.458
G4	7 (6.5%)^A,a^	7 (6.5%)^AB,a^	2 (1.9%)^A,a^	16 (14.8%)^B,C^	0.060
G5	5 (4.6%)^A,a^	6 (5.6%)^AB,a^	4 (3.7%)^A,a^	15 (13.9%)^B,C^	0.710
G6	3 (2.8%)^A,a^	3 (2.8%)^AB,a^	5 (4.6%)^A,a^	11 (10.2%)^B,C^	0.592
G7	7 (6.5%)^A,a^	5 (4.6%)^AB,a^	3 (2.8%)^A,a^	15 (13.9%)^B,C^	0.254
G8	4 (3.7%)^A,a^	5 (4.6%)^AB,a^	4 (3.7%)^A,a^	13 (12%)^B,C^	0.887
G9	7 (6.5%)^A,a^	5 (4.6%)^AB,a^	2 (1.9%)^A,a^	14 (13%)^B,C^	0.109
Total	42 (38.9%)^a^	40 (37%)^a^	26 (24.1%)^a^	108 (100%)	0.42
P Value*	0.041	0.010	0.183	< 0.001	

*Chi-square test. G1: Control; G2: Instrumentation and filling; G3: R-Endo; G4: D-Race; G5: WaveOne Gold; G6: ProTaper Retreatment; G7: Mtwo R; G8: Reciproc; G9: R1-Clearsonic. Capital letters compare groups in vertical columns and lower-case letters compare groups in horizontal rows.

## Data Availability

The datasets used and/or analyzed during the current study are available from the corresponding author.
